# BDRoad-Sense: A benchmark dataset for road surface detection and safety enhancement

**DOI:** 10.1016/j.dib.2026.112893

**Published:** 2026-05-27

**Authors:** Ali Sadman Mashrafi, Md. Abu Sayem Rabbi, Proyas Das, Md. Mujahid Alam Jihan, Md. Mahdi Hossain Hira

**Affiliations:** Department of Computer Science and Engineering, Leading University, Ragibnagar, Kamal Bazar, South Surma, Sylhet 3112, Bangladesh

**Keywords:** Road damage dataset, Multi-class classification, Data augmentation, Deep learning, Image classification, Convolutional neural network, Swin transformer, Vision transformer

## Abstract

This article presents BDRoad-Sense, a systematically collected and validated multi-class road surface anomaly dataset developed to support road safety enhancement and automated road condition classification. The dataset comprises five categories: Major Damage, Minor Damage, Normal Road, Manhole, and Speed Breaker, representing both structural road deterioration and functional infrastructure components. Image collection was conducted from November 7, 2025, to February 16, 2026, across rural and urban regions of Sylhet District, Bangladesh, using four different smartphone cameras to incorporate natural variations in imaging conditions and device characteristics. Initially, 6,350 road surface images were collected and manually screened to ensure class relevance and visual clarity. To improve dataset balance and enhance intra-class variability, controlled data augmentation techniques were applied, generating 2757 augmented images and resulting in a total dataset size of 9,107 images. All images were resized to a uniform resolution of 1024 × 1024 pixels to maintain consistency and compatibility with machine learning and computer vision frameworks. The dataset supports benchmarking of multi-class classification models, including convolutional and transformer-based architectures, and provides a structured resource for automated road monitoring, infrastructure assessment, and intelligent transportation research.

Specifications Table

**Table:** 

Subject	Computer Sciences
Specific subject area	Image Classification, Image Identification, Machine Learning, Deep Learning, and Computer Vision
Type of data	Image (.JPG)
Data collection	Image data were collected from various road locations using mobile phone cameras. Road surfaces were categorized into five classes: Major Damage, Minor Damage, Normal Road, Manhole, and Speed Breaker. Each road segment was carefully examined prior to image acquisition to ensure accurate classification based on visible structural characteristics. The captured images were subsequently annotated according to predefined class definitions to maintain consistency and reliability across the dataset. This systematic collection and labeling process ensured a comprehensive and well-structured dataset for all five road condition categories, facilitating accurate classification and benchmarking of deep learning models.
Data source location	Image data were collected from multiple road locations in Sylhet District, Bangladesh. Latitude: 24.8949° N, Longitude: 91.8687°
Data accessibility	Repository name: Mendeley Data Data identification number: 10.17632/z3nx8n396g.3 Direct URL to data: https://data.mendeley.com/datasets/z3nx8n396g/3 The dataset is released under the Creative Commons Attribution 4.0 International (CC BY 4.0) license.
Related research article	None

## 1 Value of the Data

• The dataset contains annotated road surface images categorized into five classes: Major Damage, Minor Damage, Manhole, Speed Breaker, and Normal Road. Images were collected from rural and urban regions of Bangladesh under varying illumination conditions and pavement textures, reflecting real-world road environments.
• A total of 6350 original images were expanded to 9107 images through class-specific augmentation to improve dataset balance and enhance intra-class variability. Augmentation techniques included brightness adjustment, contrast variation, and blur simulation.
• All images were resized to a uniform resolution of 1024 × 1024 pixels and organized in a structured format suitable for supervised multi-class classification tasks using standard deep learning frameworks.
• The dataset enables benchmarking of convolution-based and transformer-based models using standard evaluation metrics such as accuracy, precision, recall, F1-score, and confusion matrices, supporting class-wise performance analysis.
• The structured multi-class design supports research in automated road surface classification, road condition assessment, and infrastructure monitoring applications, particularly in rural and urban transportation environments.
• The dataset primarily benefits researchers and practitioners in computer vision, transportation engineering, and smart city planning. It can be directly used to develop AI-based road surface monitoring systems for real-time detection of road anomalies and to assist local authorities in prioritizing maintenance and repair of critical road segments, thereby improving road safety and reducing infrastructure costs.

## 2 Background

The primary motivation behind compiling this dataset was to address challenges associated with road surface deterioration and the growing demand for automated road condition monitoring systems. Road anomalies such as major damage, minor damage, exposed manholes, and irregular speed breakers significantly affect transportation safety and infrastructure maintenance planning. In developing countries, road inspection is predominantly conducted manually, making it time consuming and dependent on subjective visual assessment. Several publicly available datasets, including RDD2018, RDD2020, and RDD2022 [[1], [2], [3]], have been developed for smartphone-based road damage detection; however, they mainly focus on major and minor crack related damage. Similarly, Crack500 [4] and CrackForest [5] emphasize crack segmentation tasks, while PY-CrackDB [6] includes limited damage categories. The Dataset of Unpaved and Paved Road [7] incorporates major damage and speed breakers but does not explicitly consider exposed manholes as a separate annotated class. As illustrated in Table 7, existing datasets lack unified multi-class representation of road surface conditions. To address this limitation, the present Bangladesh dataset provides a structured collection of road surface images across five categories: Major Damage, Minor Damage, Normal Road, Manhole, and Speed Breaker, with augmentation applied to enhance class balance for supervised learning and benchmarking applications.

## 3 Data Description

The research design of the BDRoad-Sense dataset followed a structured and systematic workflow comprising data collection, preprocessing, validation, and organization. Road surface images were captured using multiple mobile cameras and categorized into five predefined classes: Major Damage, Minor Damage, Normal Road, Manhole, and Speed Breaker. Following collection, the images were screened to ensure clarity and class relevance, augmented to improve balance and intra-class variability. The detailed procedures for data acquisition, preprocessing, and validation are described in the subsequent subsections.

### 3.1 Data collection

Road surface images were manually collected from multiple locations across Sylhet District, Bangladesh, through on-site field visits covering both urban and rural road segments. Data acquisition was performed while traveling along selected roadways using motorcycles, enabling access to diverse transportation routes within the region. Prior to image capture, each road segment was visually examined to determine its surface condition. Images were collected to represent five predefined categories: Major Damage, Minor Damage, Normal Road, Manhole, and Speed Breaker. Care was taken to ensure that the primary surface feature corresponding to the designated class was clearly visible in each image. All images were captured using handheld mobile phone cameras and stored in .JPG format without additional equipment or artificial scene modification. The collection process followed consistent class definitions to maintain labeling reliability throughout the dataset. The geographical coordinates of the road image collection sites are provided in Table 1.

**Table 1:** Geographical information of road image collection sites.

Sl. No.	Location Name	Area Type	Latitude (°N)	Longitude (°E)	Image counts
1	Zindabazar	Urban	24.894922	91.868684	243
2	Ambarkhana	Urban	24.905156	91.870240	227
3	Shahjalal Upashahar	Urban	24.888248	91.885849	383
4	Akhalia	Urban	24.909598	91.841402	297
5	Sagardighir Par	Urban	24.907651	91.856420	210
6	Humayun Chattar	Urban	24.876964	91.875320	380
7	Chondipul	Urban	24.867895	91.857255	151
8	Bagbari	Urban	24.901800	91.850154	320
9	Shamimabad	Urban	24.900264	91.849217	300
10	Sheikhghat	Urban	24.888659	91.859738	283
11	Noyashorok	Urban	24.900019	91.874844	274
12	Raynogor	Urban	24.900749	91.885627	360
13	Lakkatura	Rural	24.924795	91.871289	250
14	Malnichhara	Rural	24.931932	91.866825	240
15	Shibganj	Urban	24.894951	91.890804	232
16	Kumarpara	Urban	24.897898	91.878446	218
17	Naiorpul	Urban	24.894784	91.878776	207
18	Tilagor	Urban	24.896125	91.900125	190
19	Shahporan	Rural	24.911515	91.933913	280
20	Kamal Bazar	Rural	24.881331	91.811863	420
21	Chiknagul	Rural	24.960674	92.014443	260
22	Haripur	Rural	24.990320	92.043038	250
23	Jointapur	Rural	25.140332	92.119768	375

Road surface images were acquired using four different smartphone devices to incorporate natural variation in imaging characteristics such as resolution, focal length, and sensor configuration. The use of multiple devices reflects typical mobile-based image acquisition conditions and preserves real-world variability in image quality. Detailed technical specifications of the devices used during data collection are summarized in Table 2.

**Table 2:** Details of camera specifications used for road image collection

Mobile Model Name	Resolution (MP)	Focal Length (mm)	Sensor Size (inch)	Image Captured
Apple iPhone 16 Plus	48 MP	26 mm	1/1.56	1737
Realme 11 Pro Plus	200 MP	24 mm	1/1.4	1405
Realme 10 Pro Plus	108 MP	24 mm	1/1.67	1550
Redmi Note 10	48 MP	26 mm	1/2.0	1658

During data acquisition, camera positioning parameters were not strictly controlled. Images were captured under real-world conditions using handheld smartphones.

The camera height was approximately 0.8–1.5 m, with a downward orientation toward the road surface. The distance to the road was roughly 1–3 m.

Images were taken both in stationary conditions and while moving at low speeds, preserving real-world variability and enhancing dataset robustness.

### 3.2 Data preprocessing

Following data collection, all images underwent manual screening to ensure class relevance and visual clarity. Only images in which the primary road surface condition was clearly visible were retained.

To improve class balance and increase dataset diversity, class-specific data augmentation techniques were applied, including brightness adjustment, contrast variation, and blur simulation. These transformations were performed within controlled parameter ranges to preserve the structural characteristics of each category while enhancing intra-class variability. Augmented images were stored separately from the original images to ensure transparency and reproducibility.

After augmentation, the dataset was expanded to 9107 images. All images were resized to a uniform resolution of 1024 × 1024 pixels to maintain consistency and ensure compatibility with deep learning frameworks. Table 3 presents the overall class-wise distribution of original and augmented images in the complete dataset.

**Table 3:** Distribution of original, augmented, and total dataset images across different road surface categories.

Category	Number of Original Image	Number of augmented images	Total Dataset Size
Major Damage	2062	57	2119
Minor Damage	1468	473	1941
Normal Road	1326	572	1898
Manhole	555	912	1467
Speed Breaker	939	743	1682
Total	6350	2757	9107

The ‘Number of augmented images’ column indicates only the newly generated samples and does not include original images. The total dataset size for each class is calculated as the sum of original and augmented images (Total = Original + Augmented).

The augmentation process was implemented using the OpenCV library in Python. A combination of geometric and photometric transformations was applied to enhance model generalization. Random rotations in the range of −30° to +30° were used to introduce orientation variability. Brightness and contrast jittering were applied to simulate illumination changes, while Gaussian blur was occasionally introduced to emulate mild defocus. Furthermore, hue and saturation shifts were applied to account for color variations. To improve robustness against spatial deformations, GridDistortion and elastic transformations were also incorporated.

The original dataset was first divided into three independent subsets: training, validation, and testing. To preserve class distribution across all subsets, a stratified splitting strategy was applied.

To address class imbalance, data augmentation was applied exclusively to the training subset. Augmentation was performed deterministically (probability = 1), ensuring that each selected image was augmented. Target sample sizes were set to 1500 for Major Damage, Minor Damage, and Normal Road, 1300 for Manhole, and 1400 for Speed Breaker. The number of augmented images for each class was determined based on the gap between the original training samples and these target values. All augmented samples retained their original class labels.

The final training set was constructed by combining the original training data with the augmented samples. Table 4 presents the split-specific distribution of training, validation, testing, and augmented training samples used during model development.

**Table 4:** Category-wise distribution of dataset images used for road surface classification.

Category	Original	Training Set	Validation Set	Test Set	Augmented Images	Final Training Set	Total Dataset Size
Major Damage	2062	1443	309	310	57	1500	2119
Minor Damage	1468	1027	220	221	473	1500	1941
Normal Road	1326	928	198	200	572	1500	1898
Manhole	555	388	83	84	912	1300	1467
Speed Breaker	939	657	140	142	743	1400	1682
Total	6350	4443	950	957	2757	7200	9107

### 3.3 Dataset organization and structure

**Fig. 1:**
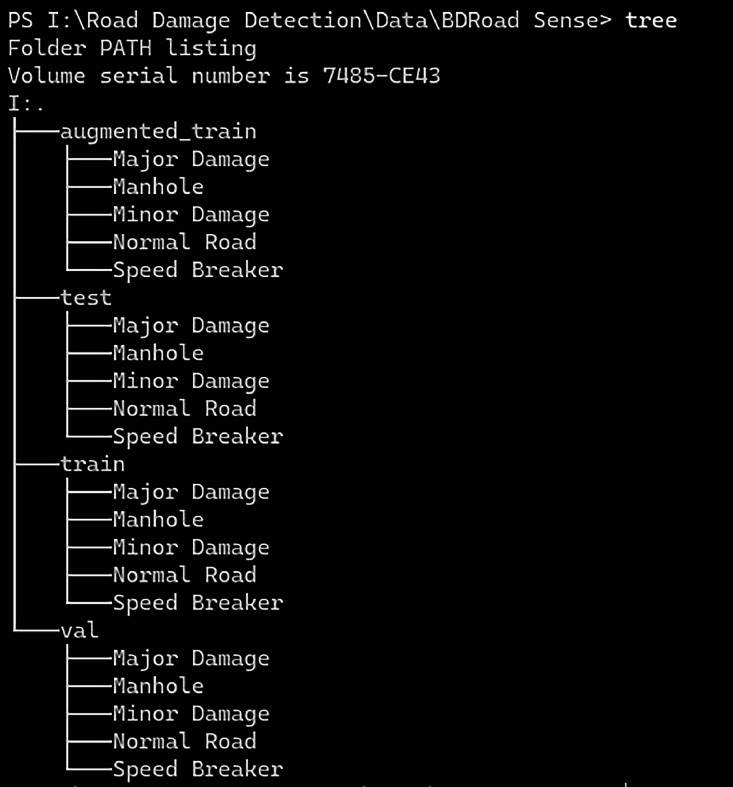
Folder structure of the dataset.

The BDRoad-Sense dataset is organized in a hierarchical directory structure, as illustrated in Fig. 1. The dataset is divided into four primary subsets: train, validation, test, and augmented_train.

Within each subset, images are further categorized into five class-specific folders: *Major Damage, Minor Damage, Normal Road, Manhole,* and *Speed Breaker*. Each folder contains only the images corresponding to its respective class, enabling efficient class-wise data loading and model training.

The augmented_train subset is generated exclusively from the original Training Set using data augmentation techniques and is utilized only during the training phase to improve model generalization and address class imbalance. The resulting Final Training Set consists of the original training images combined with the augmented images. In this study, the Final Training Set is used for model training, while the original Training Set is preserved separately for potential future research, benchmarking, and reproducibility purposes.

All images are stored in *.JPG* format and follow a sequential naming (e.g., train_major_damage_0001.jpg, test_normal_road_0001.jpg). The class labels are implicitly defined by the directory structure rather than being embedded within the file names.

The folder structure of the dataset is organized as follows:

Metadata fields are provided only for the original dataset, while augmented images inherit class labels from their corresponding original samples and do not include additional metadata.

The dataset includes structured metadata for each image in the original dataset to provide contextual information about data acquisition and labeling. Table 5 presents the metadata fields associated with each image in the original BDRoad-Sense dataset

**Table 5:** Description of metadata fields, associated data types, and corresponding values.

Metadata Field	Data Type	Values
image_path	String	Full file path of the image within the dataset directory
class	Categorical	Major Damage, Minor Damage, Normal Road, Manhole, Speed Breaker
location	Categorical	Geographic area name where the image was captured (e.g., Zindabazar, Kamal Bazar)
area_type	Categorical	Urban, Rural
device	Categorical	Apple iPhone 16 Plus, Realme 11 Pro Plus, Realme 10 Pro Plus, Redmi Note 10

These metadata fields enable researchers to filter images by class, location, or area type for subset-based evaluation, stratified analysis, and location-specific road condition monitoring.

### 3.4 Data validation and annotation

The data validation and annotation process involved careful inspection by a domain expert with a background in civil engineering and road infrastructure assessment, with annotation performed independently to ensure consistency in labeling. Following image acquisition, the expert conducted detailed visual examinations to verify road surface conditions and assign appropriate class labels, with each image annotated into a single category to maintain dataset consistency. Since the annotation was carried out by a single expert, inter-annotator disagreement did not arise. Special attention was given to distinguishing structural road damage from functional road components such as manholes and speed breakers, and the overall validation procedure is outlined in Algorithm 1 to ensure the reliability and integrity of the dataset.

**Algorithm 1:** Step by step data validation process with domain expert.

Input:	Dataset D containing N=6350 original road images across 5 classes (Major Damage, Minor Damage, Normal Road, Manhole, Speed Breaker) and domain expert E
Output:	Validated and corrected dataset D’
Step-1	Define class set C = {Major, Minor, Manhole, Speed Breaker, Normal}.
Step-2	Establish validation criteria with expert E and document as guideline T.
Step-3	Randomly sample subset Ds ⊂ D, where |Ds| = m = 0.2N ≈ 1270 images.
Step-4	For each image di ∈ Ds: - Obtain expert label E(di) - Compare with dataset label D(di) - Set ri = 1 if labels match, else ri = 0
Step-5	Compute validation accuracy: A = (Σ ri) / m
Step-6	If A < 0.95, Analyse mismatched samples, refine guideline T, and correct labels in dataset D
Step-7	Repeat Steps 3–6 for 3 iterations using different random subsets
Step-8:	Confirm validation outcome with accuracy A ≥ 0.95 and expert approval
Step-9:	Finalize validated dataset D’.

Table 6 presents the class-wise data description, including visual characteristics associated with each road surface category. The descriptions define the observable structural features used during annotation to distinguish between damage types and functional road components.

**Table 6:** Class-wise data description with visualization

Class Name	Description	Visualization
**Major Damage**	Major damage refers to severe road surface deterioration characterized by deep potholes, large cracks, surface collapse, or significant structural deformation. The damaged area typically spans a considerable portion of the lane and may include broken asphalt, exposed base layers, or loose debris. Such damage poses a high risk to vehicles and traffic safety. The image shows a severely deteriorated road section with a large eroded area, exposed soil, and broken pavement, indicating significant structural damage.	
**Minor Damage**	Minor damage includes small-scale surface defects such as shallow potholes, hairline cracks, minor surface wear, or localized roughness. These defects do not severely affect vehicle movement but indicate early-stage road deterioration that may worsen over time if left untreated. The image shows a road surface with small cracks and texture irregularities, representing minor surface wear without deep structural impact.	
**Normal Road**	Normal roads represent an intact and undamaged road surface without visible cracks, potholes, or structural defects. The surface appears smooth, uniform, and safe for regular vehicle movement under normal traffic conditions. The image shows a smooth and uniform road surface without visible defects, indicating a normal and undamaged condition.	
**Manhole**	Manhole refers to a circular or rectangular utility access cover embedded within the road surface. It may be metallic or concrete and is typically flush or slightly raised relative to the surrounding pavement. Manholes are intentional infrastructure components and should not be confused with road damage. The image shows a clearly visible rectangular metal utility cover embedded within the road surface, representing a manhole structure.	
**Speed Breaker**	Speed breaker is a raised section of the road constructed intentionally to reduce vehicle speed. It appears as a transverse elevation across the lane and may be painted or unpainted. Unlike road damage, it is a controlled traffic-calming structure. The image shows a raised bump across the road surface, indicating a speed breaker designed to reduce vehicle speed.	

Table 7 provides a structural comparison between the proposed dataset and selected existing road damage datasets. The comparison highlights differences in class coverage, number of images, and dataset scope.

**Table 7:** Comparison with available dataset on roads.

Dataset	Class	Number of original images	Number of augmented images	Damage Type Coverage (Class)				
				Major Damage	Minor Damage	Normal Road	Speed breaker	Manhole
Maeda et al. (RDD2018) [1]	4	9053	×	✓	✓	×	×	×
RDD2020 [2]	4	26336	×	✓	✓	×	×	×
RDD2022 [3]	4	47,420	×	✓	✓	×	×	×
Crack500 [4]	1	1119	×	×	✓	×	×	×
CrackForest [5]	1	118	×	×	✓	×	×	×
PY-CrackDB [6]	2	569	×	×	✓	✓	×	×
Dataset of Unpaved and Paved Road [7]	4	4242	4242	✓	×	×	✓	×
This dataset	5	6350	2757	✓	✓	✓	✓	✓

The validation process was performed on the original dataset (N = 6350), while augmented images inherit labels from the validated samples.

When multiple road surface features were simultaneously visible in a single image, the dominant class was assigned based on the spatial prominence of the feature specifically, the class whose corresponding feature occupied the largest visible area or appeared most centrally within the frame. In cases where spatial prominence alone was insufficient to resolve ambiguity, the final labeling decision was made by the domain expert based on the established annotation guideline.

These categories are grouped within a single classification dataset because they represent distinct, visually identifiable road surface conditions and features that commonly appear in real-world road scenes and are relevant for automated road understanding. Although some classes correspond to damage (Major Damage, Minor Damage) and others to infrastructure elements (Manhole, Speed Breaker), they are unified by their shared role as detectable road-level feature**s** that a vision-based system must distinguish during classification tasks.

The boundaries between classes were defined through explicit annotation criteria based on visual characteristics. Major Damage was assigned to severe defects such as deep potholes or large cracks, while Minor Damage included less severe surface irregularities. Normal Road was used when no visible defects or structures were present. Manhole and Speed Breaker were labeled whenever these specific structural elements were clearly visible, regardless of surrounding conditions. To avoid ambiguity, each image was assigned a single dominant **class** based on the most prominent feature in the scene, ensuring mutual exclusivity and consistent labeling across the dataset.

Existing datasets such as RDD2018 and RDD2020, which primarily focus on road damage, the proposed dataset provides a unified multi-class representation including both damage types and functional road elements (manhole and speed breaker) along with normal road conditions. In Bangladesh, speed breakers and manholes are often irregular (raised or depressed), posing safety risks. This dataset enables the development of automated vision-based systems capable of distinguishing these features and generating real-time road safety alerts, supporting more comprehensive and practical road monitoring applications.

## 4 Experimental Design, Materials and Methods

**Fig. 2:**
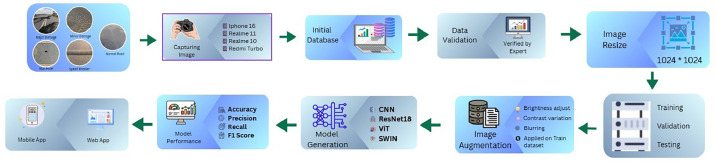
Workflow of dataset preparation and evaluation process.

The experimental design followed a structured pipeline including data acquisition, validation, preprocessing, and model evaluation. Data augmentation techniques, including brightness adjustment, contrast variation, and blur simulation, were applied to increase variability across categories. All images were resized to 1024 × 1024 pixels during preprocessing. To improve robustness and prevent data leakage, the dataset was first divided into independent training, validation, and testing subsets prior to augmentation, and augmentation was applied exclusively to the training subset. The complete workflow of dataset preparation and model evaluation is illustrated in Fig. 2.

### 4.1 Baseline architecture and experimental setup

To benchmark the dataset, four deep learning architectures were considered: a conventional CNN, ResNet18, Vision Transformer (ViT), and Swin Transformer, representing convolution-based and transformer-based approaches.

The experiments were conducted using the augmented dataset. A stratified data splitting strategy was employed to divide the dataset into training, validation, and testing subsets with a ratio of 70:15:15, ensuring that class distributions were preserved across all subsets. The splitting was performed using the train_test_split function from the scikit-learn library with a fixed random seed (random_state = 42) to ensure reproducibility. Data augmentation was applied only to the training subset to improve model generalization, while validation and testing sets were kept unchanged for unbiased evaluation.

Although augmented samples are derived from the same base images, the applied transformations (brightness and contrast adjustment, Gaussian blur) introduce sufficient variability in visual appearance while preserving class semantics.

The dataset images were originally stored at a uniform resolution of 1024 × 1024 pixels. For model training and evaluation, all input images were subsequently resized to 224 × 224 pixels and normalized using ImageNet statistics to ensure compatibility with the selected deep learning architectures. Training was performed using a batch size of 32 for CNN and ResNet18 models, and a batch size of 8 for ViT and Swin Transformer models. The models were trained for up to 25 epochs using the AdamW optimizer (learning rate = 1 × 10⁻⁴, weight decay = 1 × 10⁻⁴). Early stopping with a patience of 6 epochs was applied based on validation accuracy.

All images were normalized consistently across all models. Data augmentation was applied only as an offline preprocessing step, and no additional augmentation was used during training.

Pretrained ResNet18, ViT (base, patch16-224), and Swin Transformer (base, patch4-window7-224) models were fine-tuned for five-class classification by replacing the final classification layer. The CNN model consisted of stacked convolutional layers followed by fully connected layers with dropout regularization.

Performance was evaluated on the independent test set using accuracy, precision, recall, F1-score, confusion matrices.

Experiments were conducted on a local workstation equipped with an MSI RTX 3060 GPU (12 GB VRAM) and 16 GB RAM using Python 3.12 and PyTorch with supporting libraries.

### 4.2 Model performance

Model performance was evaluated using accuracy, precision, recall, and F1-score. The quantitative results are summarized in Table 8, while class-wise prediction behavior is illustrated through confusion matrices. Each model was trained three times using different random seeds (42, 123, and 777) to ensure robustness

**Table 8:** Comparative performance evaluation of models.

Model	Accuracy (%)	Precision (%)	Recall (%)	F1 Score (%)
CNN	73.32 ± 1.45	73.61 ± 1.51	73.32 ± 1.45	73.34 ± 1.50
ResNet18	88.72 ± 2.17	88.91 ± 1.80	88.72 ± 2.08	88.77 ± 1.96
ViT	86.80 ± 0.51	87.01 ± 0.16	86.80 ± 0.51	86.86 ± 0.37
Swin	90.67 ± 1.15	90.89 ± 0.98	90.67 ± 1.15	90.72 ± 1.09

The baseline CNN achieved an accuracy of 73.32 %, with comparable precision 73.61 % recall 73.32 %and F1-score 73.34 %. ResNet18 and ViT achieved overall accuracies of 88.72 % and 86.80 %, respectively, with consistently balanced precision, recall, and F1-score values. The Swin Transformer demonstrated the best performance, achieving 90.67 % across all reported metrics.

Values are reported as mean ± standard deviation across three runs using random seeds 42, 123, and 777.

**Fig. 3:**
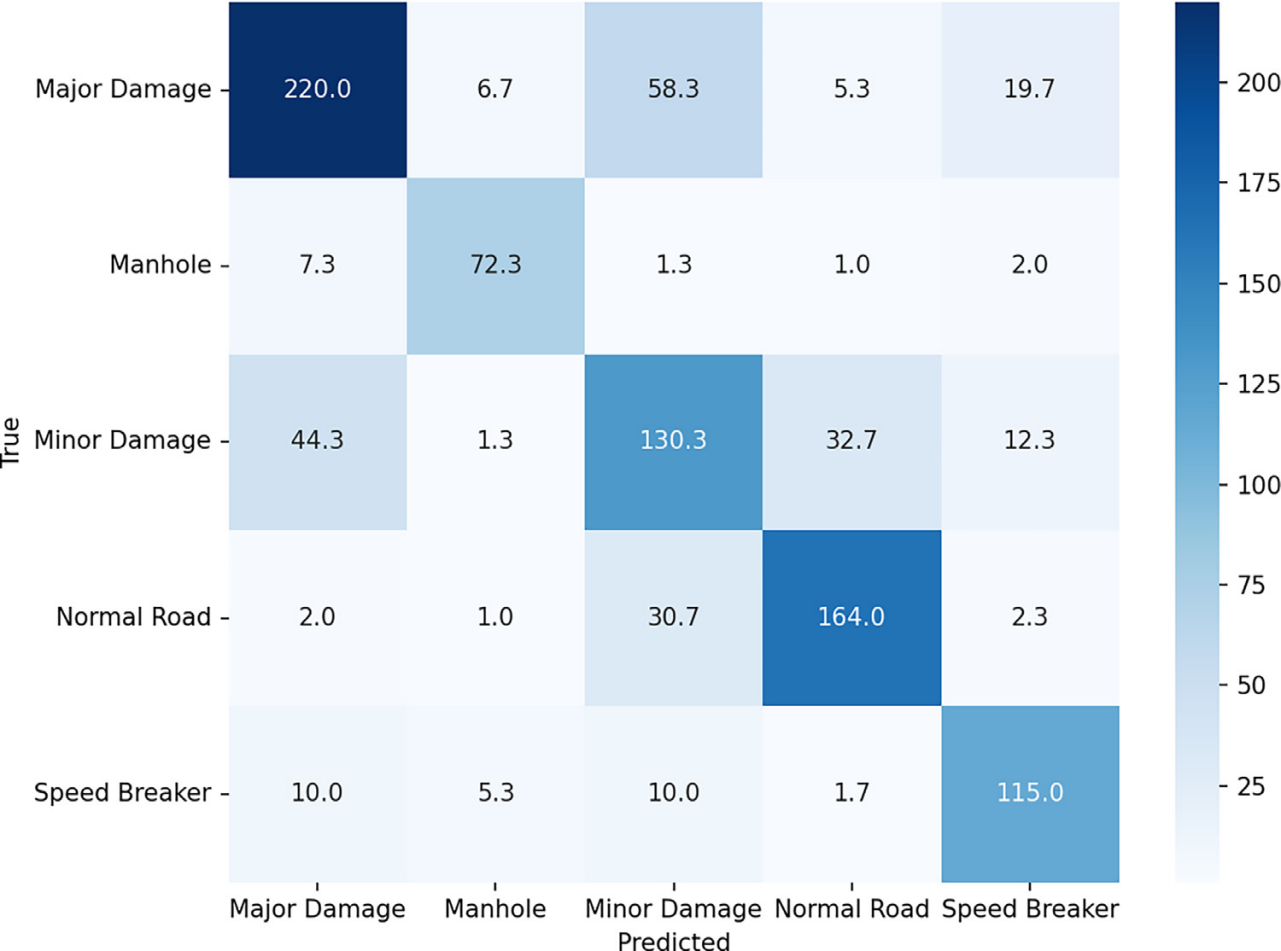
Convolutional neural network (CNN) confusion matrix.

**Fig. 4:**
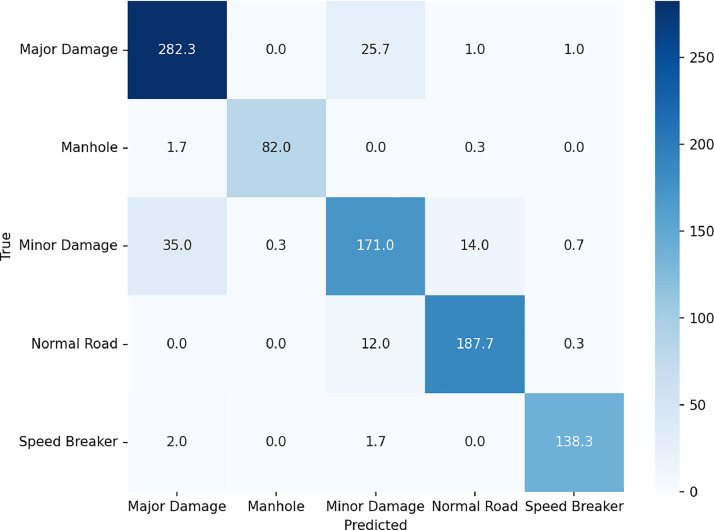
Residual network-18 layers (ResNet18) confusion matrix.

**Fig. 5:**
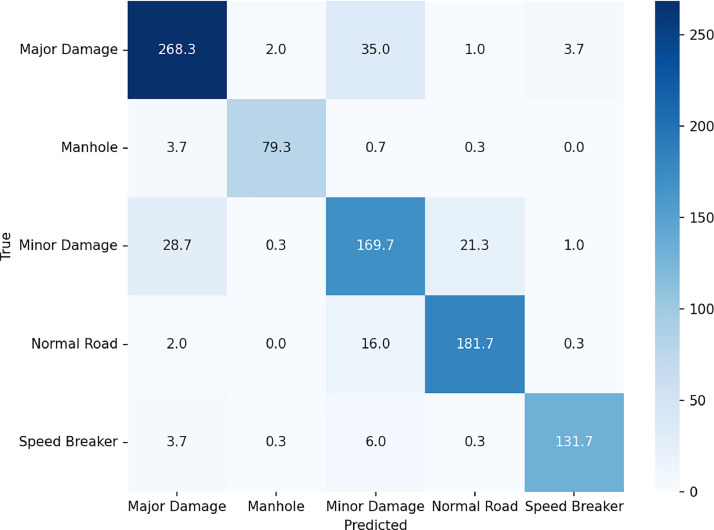
Vision transformer (ViT) confusion matrix.

**Fig. 6:**
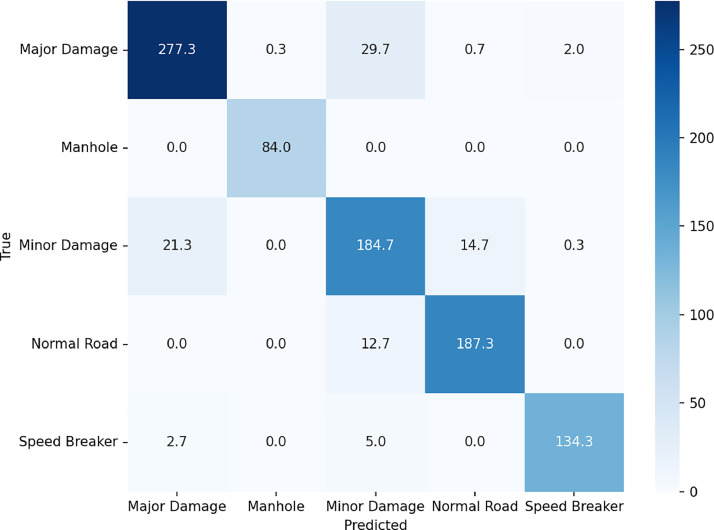
Shifted window transformer (Swin) confusion matrix.

Confusion matrix analysis demonstrates improved class separability for transformer-based architectures. The reported confusion matrices represent the average results obtained using random seeds 42, 123, and 777. The class-wise prediction distributions for each model are illustrated in Fig. 3 (CNN), Fig. 4 (ResNet18), Fig. 5 (ViT), and Fig. 6 (Swin Transformer), highlighting differences in misclassification patterns and overall prediction consistency.

The complete implementation, including image resizing procedures, data augmentation scripts, model training pipelines, and evaluation codes, is publicly available in the associated GitHub repository [8] to ensure transparency and reproducibility.

## Limitations

The dataset contains a substantial number of images across five road surface categories (Major Damage, Minor Damage, Normal Road, Manhole, and Speed Breaker). However, data collection is geographically concentrated within Bangladesh, which may limit direct generalization to regions with different construction materials or environmental conditions.

Images were captured using standard mobile camera devices under varying lighting and weather conditions. While this reflects real-world scenarios and enhances practical relevance, such variations may introduce inconsistencies that could influence model performance in highly controlled environments.

The dataset focuses on surface-level road anomalies and does not include subsurface structural information. Additionally, temporal progression data were not collected, limiting longitudinal analysis of damage evolution.

## Ethics Statement

We confirm that the authors have read and followed the ethical requirements for publication in Data in Brief and confirming that the current work does not involve human subjects, animal experiments, or any data collected from social media platforms.

## CRediT Author Statement

**Ali Sadman Mashrafi:** Data curation, Methodology, Software, Writing – original draft; **Md. Abu Sayem Rabbi:** Data curation, Methodology, Software, Writing –Review & Editing; **Proyas Das:** Data curation, Visualization; **Md. Mujahid Alam Jihan:** Data curation, Visualization; **Md. Mahdi Hossain Hira:** Supervision, Writing –Review.

## Data Availability

Mendeley DataBDRoad-Sense: A Benchmark Dataset for Road Surface Detection and Safety Enhancement (Original data).
